# Epidemiology of Postherpetic Neuralgia in Korea: An Electronic Population Health Insurance System Based Study

**DOI:** 10.1097/MD.0000000000003304

**Published:** 2016-04-08

**Authors:** Myong-Joo Hong, Yeon-Dong Kim, Yong-Kwan Cheong, Seon-Jeong Park, Seung-Won Choi, Hyon-Joo Hong

**Affiliations:** From the Department of Rheumatology (M-JH), Presbyterian Medical Center, Jeonju; Department of Anesthesiology and Pain Medicine, Wonkwang University Hospital, School of Medicine (Y-DK, Y-KC, S-JP, S-WC), Institute of Wonkwang Medical Science (Y-DK), Iksan; and Department of Nursing (H-JH), Graduate School, Kyung Hee University, Seoul, Republic of Korea.

## Abstract

Postherpetic neuralgia (PHN) is a disease entity defined as persistent pain after the acute pain of herpes zoster gradually resolves. It is associated with impaired daily activities, resulting in reduced quality of life. General epidemiological data on PHN is necessary for the effective management. However, data on the epidemiology of PHN in Korea is lacking. The aim of this study was to evaluate the epidemiological features of PHN in the general population.

We used population-based medical data for 51,448,491 subscribers to the Health Insurance Service in the year of 2013 to analyze of PHN epidemiology in Korea, such as the incidence, regional distribution, seasonal variation, and healthcare resource utilization. Total number of patients and medical cost on PHN were analyzed from 2009 to 2013.

Findings indicate that the incidence of PHN in Korea was 2.5 per 1000 person-years, which was strongly correlated with age and sex. There were no differences in seasonal variation or regional distribution. The medical cost increased steadily over the study period. When admitted to general hospitals, patients with PHN were mainly managed in the dermatology and anesthesiology departments.

The incidence and prevalence rates of PHN in Koreans appear to be considerably higher compared to those in western populations, while the sex and age predisposition was similar. Considering that the pain associated with PHN can have a marked impact on a patient's quality of life resulting in a medicosocial economic burden, anesthesiology physicians have an important role in primary care in Korea. Future research on the cost-effectiveness of the management of PHN is needed.

## INTRODUCTION

Herpes zoster (HZ), clinically known as shingles, is caused by the reactivation of the varicella zoster virus (VZV) in the neurons of the cranial nerve ganglia or dorsal root ganglia along the entire neuroaxis after primary infection with varicella (chickenpox), which usually occurs in childhood. It is characterized by a vesiculobullous rash along the skin dermatomes, and is accompanied by severe pain.^1^ In Patients suffered from HZ, postherpetic neuralgia (PHN) is considered to be the most common complication of the disease. Even though a few, minority patients with HZ develop the severe form of PHN, it is worthy of attention in clinical practice. There is no clear for definition for PHN, either on duration or degree of the pain. Many physicians generally consider it as PHN if the time of pain persists more than 3 months after developing skin vesicles, and some consider it after developing pain.^2^

The incidence of HZ remains very common with a 23% to 30% lifetime risk.^3,4^ Although several epidemiological studies on HZ in Korea have been conducted,^5^ data on the epidemiology and costs related to PHN in Korea are lacking. To our knowledge, there has only been 1 observational study conducted to investigate PHN in Korea; the study depended on limited hospital-based data and focused on the treatment status. Therefore, the results may not be representative of the characteristics of PHN across the Korea.^6^

The management of pain related to PHN, with pharmacologic or nonpharmacologic methods, is often considered to be suboptimal.^7^ PHN-associated pain can significantly interfere with all activities of daily livings (ADLs), particularly mood, general activities, and sleep. It can also affect healthcare resource utilization (HCRU) associated with the management of PHN, including visits to the primary care doctor, a specialist or a physiotherapist, hospitalizations, and visits to the emergency department leading to an increased economic burden. As Korea has a trend toward a rapidly increasing elderly population,^8^ PHN has societal implications because of its high prevalence in elderly people and economic burden on the national healthcare system. Recent research has demonstrated that a VZV vaccine could substantially reduce the incidence of HZ and resulting complications.^9^ In order to understand the potential impact of a vaccine and the efficacy of other treatment options, data related to the epidemiology and burden of PHN in Korea are required.

The purpose of this study was to evaluate the incidence and other epidemiological features of PHN, including HCRU, in Korea. These data will be valuable to inform the evidence-based decision-making process related to the management of PHN.

## MATERIALS AND METHODS

The Institutional Review Board of Wonkwang university hospital approved this study (IRB no. 201508HRE081); the need for informed patient consent was waived. In Korea, all citizens have been covered by the National Health Insurance Service (NHIS) since 1989. It is the only public medical insurance system in Korea, and it covers 100% of the population. It is based on the subscribers’ residential status, which reflects the prevalence of a disease entity by region. All demographic data, including age and sex, are collected by the NHIS according to each citizen's Korean identification (ID) number. The Health Insurance Review and Assessment Service (HIRA), founded in 2000, computerized its date in 2005, is a government incorporated organization that was developed for medical billing purposes at the NHIS which is the only public medical insurance system in Korea, and covers 100% of the population. All medical records including diagnoses, physical and laboratory examinations, treatments, prescriptions, and hospitalizations held by medical delivery system and medical departments by specialty subject are documented in a computerized database along with the individual's Korean ID number. Current medical delivery system in Korea has been distinguished into 3 types: primary healthcare facilities (PHCFs), hospitals, and general hospitals since 1989. PHCFs are usually outpatient based clinics including all medical specialities, even with general and family physicians, whereas hospitals and general hospitals are held to higher standards by law and are capable of treating patients with more severe illnesses. Hospitals and general hospitals are divided according to medical specialities and number of hospital beds. The number of hospitals including primary clinic, hospital, and general hospital in Korea was 34,569 in the year of 2013.

On the basis of these data, we analyzed the incidence, regional distribution, seasonal variation in 2013. Total number of patients and nominal medical cost were calculated through, 2009 to 2013, for the analysis of means and trends.

### Study Population

Our calculations of the patients of PHN were based on data from 51,448,491 medical insurance subscribers of HIRA from in the year of 2013. The incidence, seasonal variation, and regional distribution of PHN patients were calculated from the HIRA and national population data, the national residence registration data census, Korean Statistical Information System in Statistics Korea. Cases were counted, on the day of diagnosis by the physicians. The number of overlapped patients was removed from the data.

### Case Definition of PHN

PHN cases were identified through a database search for any subjects with a domestic HIRA code for PHN (G53.0) more than twice as either a primary or secondary diagnosis, which is based on the World Health Organization International Classification of Diseases (ICD-9) code, clinically pain persisting or recurring 3 months after the onset of HZ.

### Statistical Analysis

The average incidence per 100,000 Korean individuals was calculated using the number of PHN cases and population data reported by the National Statistical Office of South Korea (http://kosis.kr) in 2013. All results were produced using SPSS, version 20.0 (IBM SPSS, Inc., Chicago, IL) for statistical analysis. All variables were described by number or percentage.

## RESULTS

### Incidence of PHN

**FIGURE 1:**
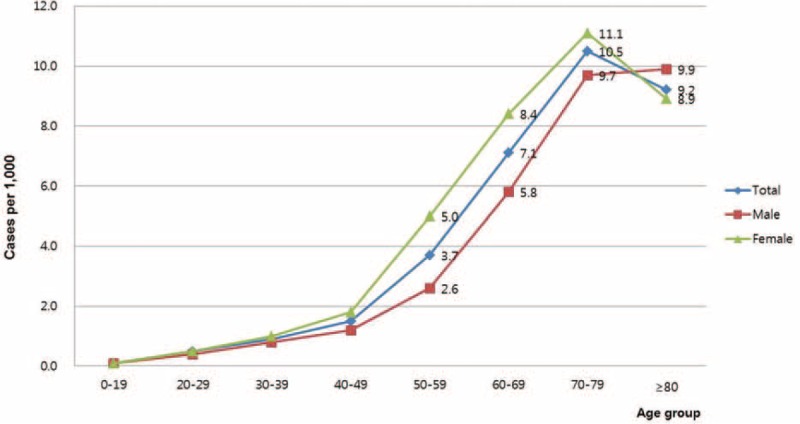
Incidence of postherpetic neuralgia in Korea in 2013.

In total, 127,657 individuals with PHN were identified in 2013 (119,390 in 2012, 103,577 in 2011, 90,706 in 2010, and 80,389 in 2009) corresponding to an overall incidence rate of 2.5 per 1000 person-years. The incidence was strongly correlated with age, with more than 50% of the patients over 60 years old. The peak PHN incidence (10.5 per 1000 person-years) was observed in patients aged 70 to 79 years. The incidence rate was higher in women than in men (3.1 per 1000 person-years in women vs 1.9 per 1000 person-years in men). Based on age-specific incidence data, this trend was observed across all age groups, especially in those patients over 50 years old (Figure 1).

### Seasonal Variation of PHN

PHN cases were categorized according to the season of occurrence. Of all PHN cases, 23.3%, 24.4%, 26.7%, 25.6% occurred in spring (29,744 in March, April, and May), summer (31,148 in June, July, and August), fall (34,020 in September, October, and November), and winter (32,745 in December, January, and February), respectively. A trend in seasonal variation in PHN cases was not observed from month to month in 2013.

### Regional Distribution of PHN

**FIGURE 2:**
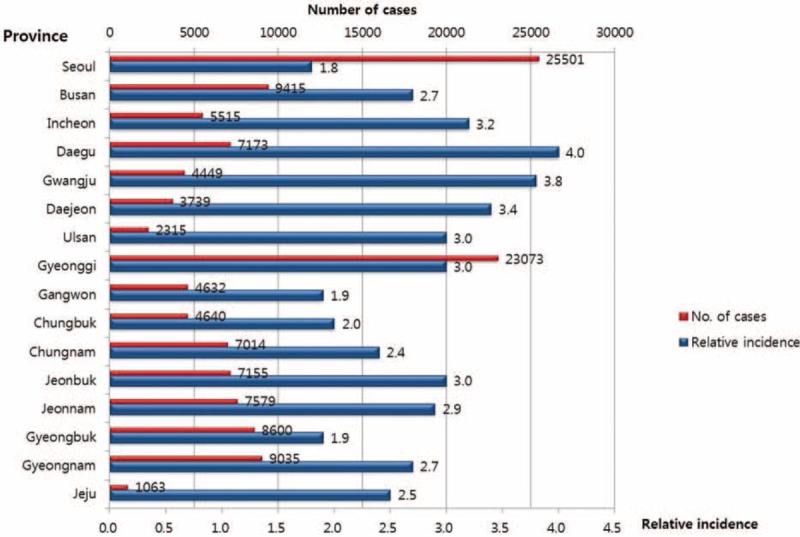
Regional distribution of postherpetic neuralgia in Korea in 2013.

To calculate the relative incidence of PHN by region (province), the population density of each province in 2013 was considered. Additionally, age-stratified random sample of 127,657 PHN patients to gather more detailed descriptive epidemiology statistics. Figure 2 shows the number of PHN cases by province and the relative incidence of PHN. The difference in relative incidence between provinces was not existed.

### Total Number of Patients and Medical Cost of PHN

**FIGURE 3:**
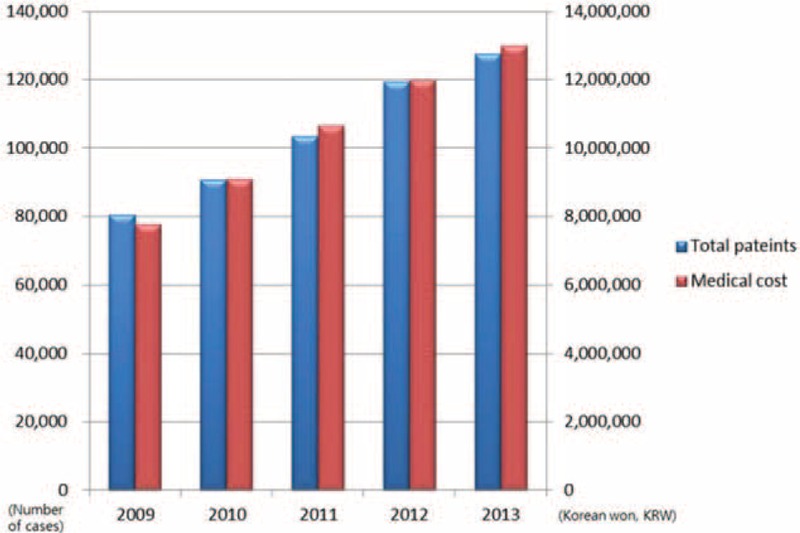
Total number of patients and medical cost of postherpetic neuralgia from 2009 to 2013.

From 2009 to 2013, the total number of patients and medical costs grew steadily. In 2013, the total number of patients had increased by approximately 58%, and medical costs by approximately 40% compared to 2009 (Figure 3).

### HCRU by Medical Delivery System and Medical Specialty

According to NHIS data from 2013, PHN patients generally (76.5% of patients) visited PHCFs for treatment. Hospitals and general hospitals were visited 16.7% and 6.8% of the time, respectively. The average number of visits per patient was 3.8 for the general hospitals and 4.7 for hospitals compared to 1.0 for PHCFs (Table 1).

**TABLE 1:**
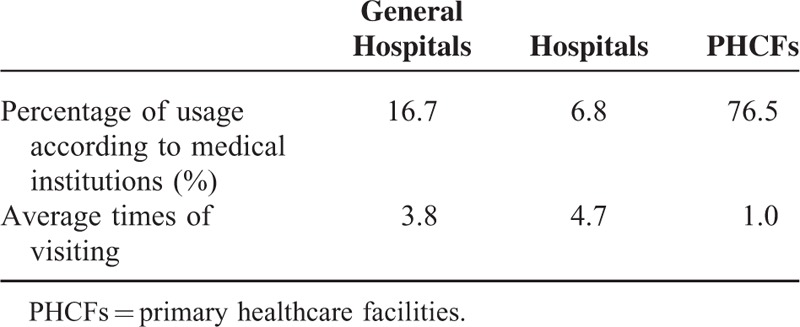
Healthcare Resource Utilization by Medical Institution and Average Times of Visiting in Patients With Postherpetic Neuralgia in 2013

**FIGURE 4:**
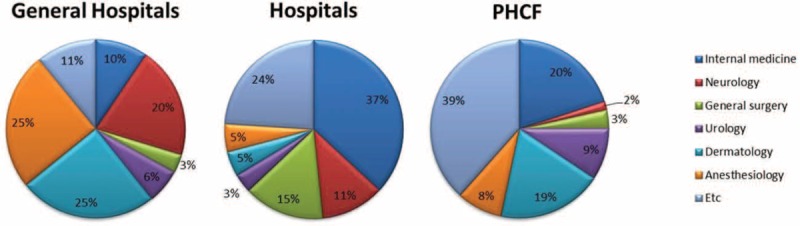
Healthcare resource utilization of patients with postherpetic neuralgia in 2013 by medical specialty. PHCF = primary healthcare facilities.

When stratified by medical specialty, in the PHCFs, patients with PHN usually presented to internal medicine (20%) and dermatology (19%) physicians (Figure 4). Similarly, patients were treated in the internal medicine departments in hospitals (37%). However, most patients who presented at general hospitals were managed in the dermatology or anesthesiology departments (both 25%).

## DISCUSSION

The syndrome of PHN is defined solely by the persistence of pain after HZ. Patients with PHN suffer from pain characterized with constant aching, burning, lancinating pain, and allodynia. The pathophysiology of PHN remains unclear. However, pathologic studies have demonstrated damage to the sensory nerves, the sensory dorsal root ganglia, and the dorsal horns of the spinal cord in patients with this condition. Indeed, ongoing inflammation has been suggested as one of the mechanisms that uphold pain in PHN.^10^

This is the first epidemiological study focused on PHN in Korea based on electronic population health insurance system data. The results of this study provide a real evaluation of medical costs and the patterns of HCRU related to PHN that ever did not analyzed. The incidence of PHN in Korea was 2.5 per 1000 person-years, which was strongly correlated with age. Most of the previous reports on the incidence of PHN have considered cases that had developed from HZ. One study, which analyzed PHN in a general practice population (3600–3800 patients) for 26 years from 1947 to 1972 in the United Kingdom, reported that PHN occurred at a rate of 0.49 per 1000 person-years.^11^ Our study shows a much higher incidence of PHN. As the pathophysiology of PHN is considered a persisting course owing to HZ, its incidence has a close relationship to that of HZ. Based on a recent report, the incidence of HZ in Korea is also higher than that of other countries, such as the United States, Canada, and South America, with an incidence of 10.4 per 1000 person-years compared to 4 to 4.5 per 1000 person-years.^12^ The higher incidence of PHN found in our study compared to the UK study could also be explained by differences in incidence rates over time. From 2000 to 2008, there was an increase in the incidence of HZ (from 28% to 54.8%).^13,14^

An aging population and the increased prevalence of chronic diseases associated with immunocompromised states in Korea could also be contributing to the observed increased incidence. By 2050, it is expected that Korea will have the world's second oldest population with 35% of its population over 65 years of age.^8^ Changes in the insight of both patients and physicians in the management of PHN could be an additional contributing factor. In our study, PHN occurred more often in women (3.1 per 1000 person-years) than in men (1.9 per 1000 person-years), and this difference was consistent across age groups. This may be explained by two factors. First, HZ shows a higher incidence in women.^12^ Second, a prominent feature of the medical culture in Korea is that, within the elderly population, women tend to utilize more medical services compared to men. According to data from the Korean Statistical Service in 2011, the rate of medical service use per person was 25.1 per year in men over 50 years old and 33.8 per year in women.^15^ The distribution of PHN by region or season did not vary. A lack of a difference between provinces is logical as it reflects homogenous ethnic groups.

We also analyzed PHN-related medical costs and HCRU. The usage of PHCFs was remarkably higher than other institutions with 76.1% of patients visiting these facilities; this was followed by general hospitals (16.7%) and hospitals (6.8%). However, patients who presented to the general hospitals visited more often, with the average number of visits to a general hospital at 3.8, compared to 1.0 for PHCFs. Compared to patients with HZ (98.7%),^16^ the role of the PHCFs in the management of PHN was much lower. This may be due to the severity of PHN, where the pain is too severe to be managed in the primary care setting. When patients presented to PHCFs, they were most commonly managed by internal medicine. While in the general hospitals, patients with PHN were mainly managed in the dermatology and anesthesiology (25%) departments. The large proportion of patients managed in the anesthesiology department is probably because of the need for pain management in severe PHN cases. There seems to be still a lack of the recognition of the role of anesthesiology and pain medicine in the management of PHN in primary care.

A 40% increase in medical costs was observed in 2013 compare to 2009. However, the medical costs shown in this study only reflect the costs for a basic consultation. As additional costs for diagnosis, nerve blocks for pain management, and prescription drugs were not include, these results could not reflect whole management-related cost. Thus, the actual medical costs will be much higher. As reported by another study, the level of severity, including pain, in PHN will also drive the cost.^17^ Patients with severe pain were shown to incur costs 2 to 3 times higher compared to patients with mild pain,^17^ implying an economic burden of PHN.

There are some limitations of this study. First, there are no definitive diagnostic criteria for PHN. In this study, we defined PHN as consistent pain for more than 3 months after confirmed HZ. Physicians usually try to follow this definition and use objective measures, however, in Korea, PHN is often diagnosed by physician's subjective opinions not based on diagnostic criteria for time course, but based on the clinical symptoms which yield the possibility of over-diagnosis. Second, we were unable to analyze PHN by infected area of body because we do not have access to a proper diagnostic term or impression code by the part of body in Korea, unlike for complex regional pain syndrome. Thirdly, compared to western countries, analysis of the rate of PHN development from HZ in Korea is difficult because of the lack of a connected system across the entire medical service and frequent changes of hospital patients. Therefore, it is not even easy to estimate the severity of PHN because of limited data such as the hospitalization rate at onset. Last, data on predisposing factors and comorbidities disease such as a diabetes, malignancy, or other immunocompromised state of patients could not be available, which needs more study.

The purpose of this study was to analyze the epidemiology of PHN in Korea, and to evaluate the medical costs and HCRU associated with the management of PHN. The incidence and prevalence rates of PHN in Koreans appear to be considerably higher compared to those in western populations, while the sex and age predisposition was similar to those observed western populations. Considering that the pain associated with PHN can have a marked impact on a patient's quality of life and limit ADLs,^18^ resulting in a considerable medicosocial economic burden, anesthesiology physicians have an important role in primary care in Korea. In addition, as PHN is associated with a rapidly aging society in Korea, future research on the predisposing factors, transition rate form HZ and cost-effectiveness of the management of PHN, including studies on interventional and noninterventional treatment methods and vaccination are needed.
